# Epidemiologic analysis and mortality outcome of firearm injuries in French Guiana (2016–2019)

**DOI:** 10.1007/s00068-024-02499-7

**Published:** 2024-03-21

**Authors:** Alexis Fremery, Elliott Beguinot, Angélique Franchi, Mathilde Douchet, Victor Tertre, Karim Hamiche, Antoine Adenis, Jean Marc Pujo, Hatem Kallel

**Affiliations:** 1 Emergency Department, Cayenne General Hospital, French Guiana, France; 2 French Guiana University, French Guiana, France; 3 Forensic Medical Unit, Cayenne General Hospital, French Guiana, France; 4 CIC INSERM1424, Cayenne General Hospital, French Guiana, France; 5 Intensive Care Unit, Cayenne General Hospital, French Guiana, France

**Keywords:** Ballistic trauma, Gunshot wounds, French Guiana, Epidemiology, Firearm injuries

## Abstract

**Background:**

French Guiana (FG) is a French territory located in South America with the highest rate of armed assaults. FG presents a poorly developed road system and a young and precarious population that makes the geographical and socio-demographic characteristics specific. No data concerning the firearm injury management are available in this country. Studying thesis trauma could permit to improve the management of victims. The objective of this study is to investigate the epidemiology of firearm injuries in FG, to define characteristics of the victims, and to assess factors associated with death. These identified factors could enable target primary prevention and intensification of medical management.

**Methods:**

From January 2016 to December 2019, we conducted a retrospective study at the Cayenne General Hospital (CGH), including all patients admitted for firearm injuries in the emergency department, the medical emergency and resuscitation service, and the forensic service. A bivariate analysis was performed to assess relevant clinical data that were entered into a logistic regression model to assess factors associated with death.

**Results:**

A total of 871 files were analyzed concerning 340 patients included after cross-checking. Victims were mainly males (90%) and young (30 ± 11 years old). The injury occurred mainly at night (60%), in a context of assaults (83%) and with long-barreled guns (82%). Among the 290 patients managed at the CGH, 60% were hospitalized including 12% that were in the intensive care unit, 41% that required surgical treatment, and 7% that died in hospital. The overall average length of stay was 10 ± 18 days. Overall mortality (*n* = 71, 21%) is statistically associated with male gender (*p* = 0.007) and suicide context (*p* < 0.001). In multivariate analysis, the sites of wounds (head and neck, thorax; *p* < 0.001) as well as induced organ injuries (neurological, respiratory, and vascular; *p* < 0.005) were independent factors associated to mortality.

**Conclusions:**

This work underlines the high incidence of ballistic trauma in FG. This mainly involves a young and male population linked to the use of long arms and assaults. Despite the geographical difficulties of the territory and the technical platform deficits (no neurosurgery, no cardiothoracic surgery, no interventional radiology), the mortality is comparable to other studies, but remains more than twice as high as in mainland France. Finally, despite a change in legislation restricting access to firearms, our results show that gunshot firearm injuries remain a major public health concern requiring greater political actions.

## Introduction

Firearm injuries are a major public health concern: 251,000 associated deaths worldwide in 2016 (3.4 deaths/100,000 persons per year) [1]. Currently, up to 875 million weapons are in circulation in the world, mostly in civilian hands (75%) [2]. Ballistic traumas cause acute injuries and chronic sequelae responsible for many disabilities [3]. They mainly concern a young population and are significantly care-consuming [1]. Some studies have identified low socio-economic status and ownership of firearms as the main risk factors for firearm-related deaths [4]. In France, firearm-related deaths represent 5.5% of traumatic deaths [5].

French Guiana is a French overseas territory located in South America, bordering Suriname and Brazil. In 2017, there were 268,700 inhabitants with a high-density population on the coastline and nearly 30% living in Cayenne, the capital [6]. The healthcare network presents three level 3 trauma centers located in the three main coastal towns (Cayenne, Kourou, and Saint-Laurent-du-Maroni) and 17 prevention and care centers located in remote areas [7, 8]. The main hospital is located in Cayenne where the intensive care unit, the major surgery specialties, and the emergency medical service can be found. But there was no neurosurgery, no cardiothoracic surgery, and no interventional radiology during the study period [9]. The nearest French level 1 trauma center is in the French West Indies, at 1500 km from Cayenne. These different parameters translate the difficulties in the management of severe injured victims.

French Guiana is the French territory with the highest rates of crime and violence [10]. Given its geography, French Guiana is a hotspot for illegal practices such as drug trafficking [11] and gold panning [12], generating a climate of violence. In 2018, there were an estimated 2.7 victims of gun violence assaults per 1000 inhabitants in French Guiana compared to 0.2 per 1000 in mainland France [13]. The homicide rate (1.3‰ inhabitants) is ten times higher than the national average [14]. In addition, Guianese population presents a high level of precariousness and strong social inequalities [15]. It is noteworthy that the authorization of weapon possession in French Guiana is different from the national legislation. Indeed, the weapons’ sale was free (without permit) until 2017 [16] and remains nowadays more flexible than in mainland France. There are an estimated 55,000 weapons circulating in French Guiana (one weapon per six inhabitants) [2]. To date, there is no study evaluating the epidemiology and the management of ballistic trauma in French Guiana. Precise knowledge of the region’s problem could permit to develop policy decisions and public prevention messages [17] and improving skills, care, and technical management resources [18], but also the creation of care structures for victims.

We therefore conducted a retrospective study at the Cayenne General Hospital (CGH) including all firearm injury victims. The primary objective was to describe the epidemiological profile and the initial management of victims. The second objective was to assess the outcome and the factors associated with death.

## Material and methods

### Study design and population

This is a retrospective, descriptive study conducted over a 4-year period (January 1st, 2016, to December 31st, 2019). This study was conducted before the SARS-COV period to avoid bias on epidemiologic data during the epidemic. We included all firearm wound victims managed by the mobile emergency medical service, the emergency department (ED), or the forensic unit (FU) of the Cayenne General Hospital. The non-inclusion criteria were stab wounds, non-ballistic firearm wounds, weapon wound older than 7 days, and decline to participate.

### Data collection

Data were collected from the CGH computerized ED software, DMU Net® (Atos, Bezons, France), for all patients admitted with diagnoses related to a ballistic wound on International Statistical Classification of Diseases (ICD-10): W32-34, X72-74, X93-95, Y22-24. These data were crossed with the FU cases extracted from the victimology records and deceased victims’ body examinations. Hospitalization records, imagery, and biology were verified. We collected the victims’ demographic variables, the circumstances of the event, the parameters recorded during the pre-hospital period, the duration of hospitalization, and the status when victims leave the hospital (alive or dead). The criteria of severity were used as defined in the national guidelines for treatment of hemorrhagic shock: death, cardiopulmonary arrest, shock, shock index > 1, use of catecholamines, and the need for fluid infusion (> 1000 mL of intravascular solution) or mechanical ventilation during initial care. Data collection was completely managed by medical personnel, supervised by medical investigator trained in research and statistical analysis, which minimized missing data due to the retrospective protocol.

### Statistical analysis

We created a data file with anonymized patient’s information and performed descriptive analysis using Excel® (Microsoft Corp., Redmond, WA, USA) and IBM® SPSS® Statistics for Windows, version 24 (IBM Corp., Armonk, NY, USA). Continuous variables are expressed as mean ± standard deviation and compared using the Student *t* test. A Mann–Whitney *U* test was performed in case the population size was insufficient. The retrospective aspect of our study did not allow us to collect a certain amount of data. Some poorly completed or insufficiently accurate records may compromise the reliability of certain results. As our main objective was epidemiological, we decided to exclude missing data from the analyses. Categorical variables are expressed as numbers (percentages and 95% confidence intervals) and compared by the *χ*^2^ test. A Fisher exact test was used for small samples. Time was expressed as hours and minutes. Non-redundant variables selected by bivariate analysis (*p* ≤ 0.05) and considered clinically relevant were entered into the logistic regression model. Results are expressed as odds ratios (OR) with 95% confidence intervals (CI). All statistical tests were two-tailed, and *p* ≤ 0.05 was considered significant.

### Ethical and regulatory approval

This retrospective research was conducted according to the national reference methodology MR-004 (Research Not Involving the Human Person) of the National Commission on Information Technology and Liberties (CNIL) on July 16, 2018 (registered n°2224124v0), which frames the processing of personal data for study, evaluation, or research to which the Cayenne General Hospital has committed to comply according to the standards in force in France. The data were pseudonymized and processed by healthcare staff in the emergency department (principal investigator or any person under his responsibility). In addition, participants were collectively informed by posters in the emergency department, in the welcome booklet, and on the hospital website (general information on clinical research). Any objections by patients to participating in the study were considered and patients were then excluded. A collective information was made and this research was registered in the hospital internal treatment database and also on the national Health Data Hub under the number F20210505152609.

## Results

### Description of the general population

**Fig. 1:**
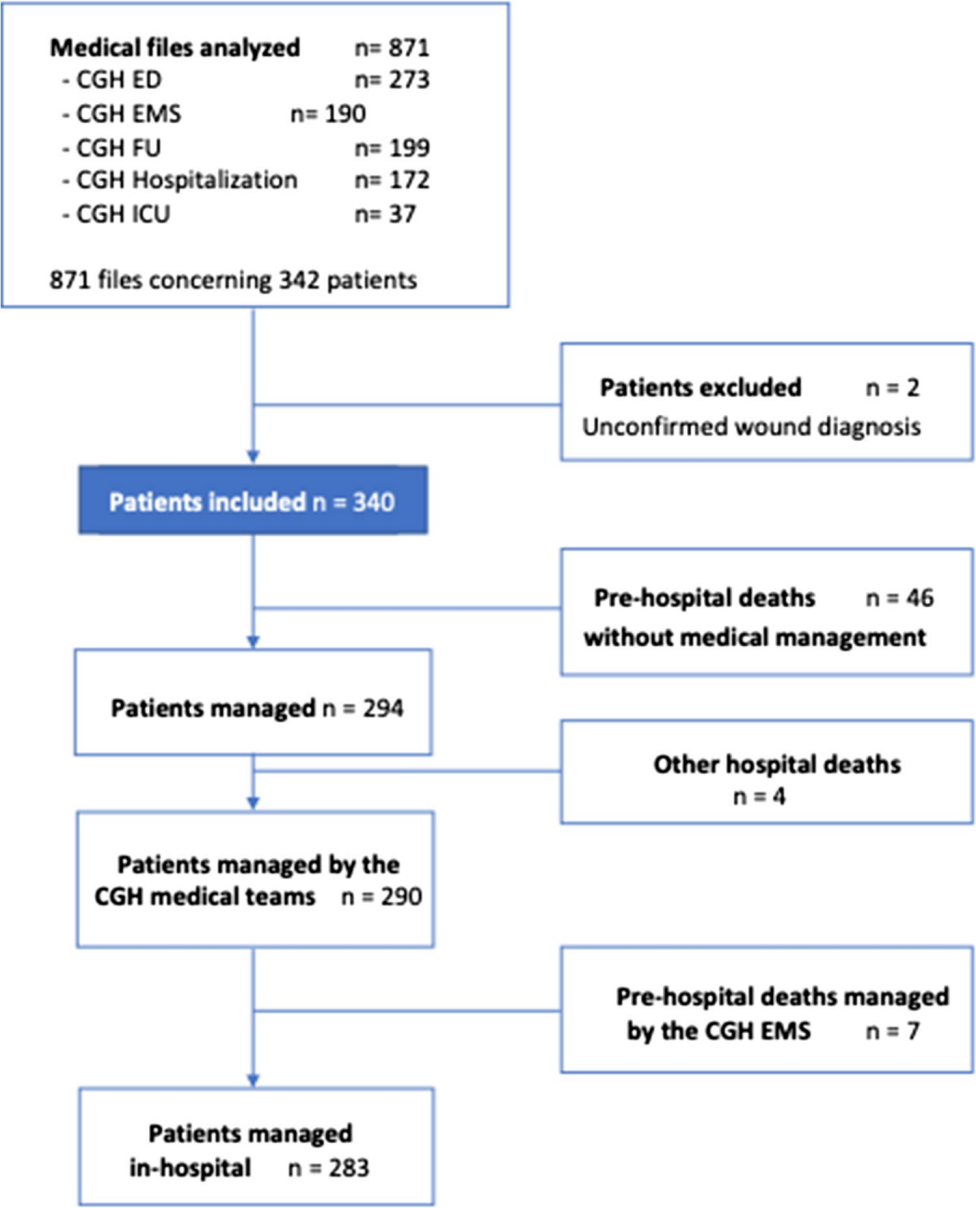
Study flow chart. CGH Cayenne General Hospital, ED emergency department, EMS emergency medical service, FU forensic unit, ICU intensive care unit

During the study period, we included 340 firearm wound victims. The flow chart of the study is reported in Fig. 1.

Demographic characteristics and circumstances of events are described in Table 1. Our data revealed a sex ratio of M/F 9:1 with an age of 30 ± 11 years. The events involved long-barreled guns in 82% and an assault context in 83% of cases.

**Table 1:** Demographic characteristics and incident circumstances

Variables	Victims (*n* = 340) (mean or percentage)	CI 95% or SD
*Demographic characteristics*		
Age *(years) (n* = *334)*	30	± 11
Sex *(male) (n* = *340)*	306 (90%)	86–93%
< 18 years old	32 (10%)	7–13%
*Incident circumstances*		
Day time (*n* = 221)		
Night (8:00 PM–8:00 AM)	132 (60%)	53–66%
Context (*n* = 318)		
Assault Accident Suicide Police arrest	265 (83%) 34 (11%) 13 (4%) 6 (2%)	79–87% 7–15% 2–7% 0.8–4%
Type of weapon (*n* = 108)		
Hand gun Long-barreled gun	19 (18%) 89 (82%)	11–27% 74–89%
Type of projectile (*n* = 314)		
Bullets Rifle leads	154 (49%) 160 (51%)	44–54% 45–56%
Shooting distance (*n* = 116)		
Far-range shooting Close-range shooting In-contact shooting	36 (31%) 65 (56%) 15 (13%)	23–40% 47–65% 8–20%

*CI* confidence interval, *SD* standard deviation

**Fig. 2:**
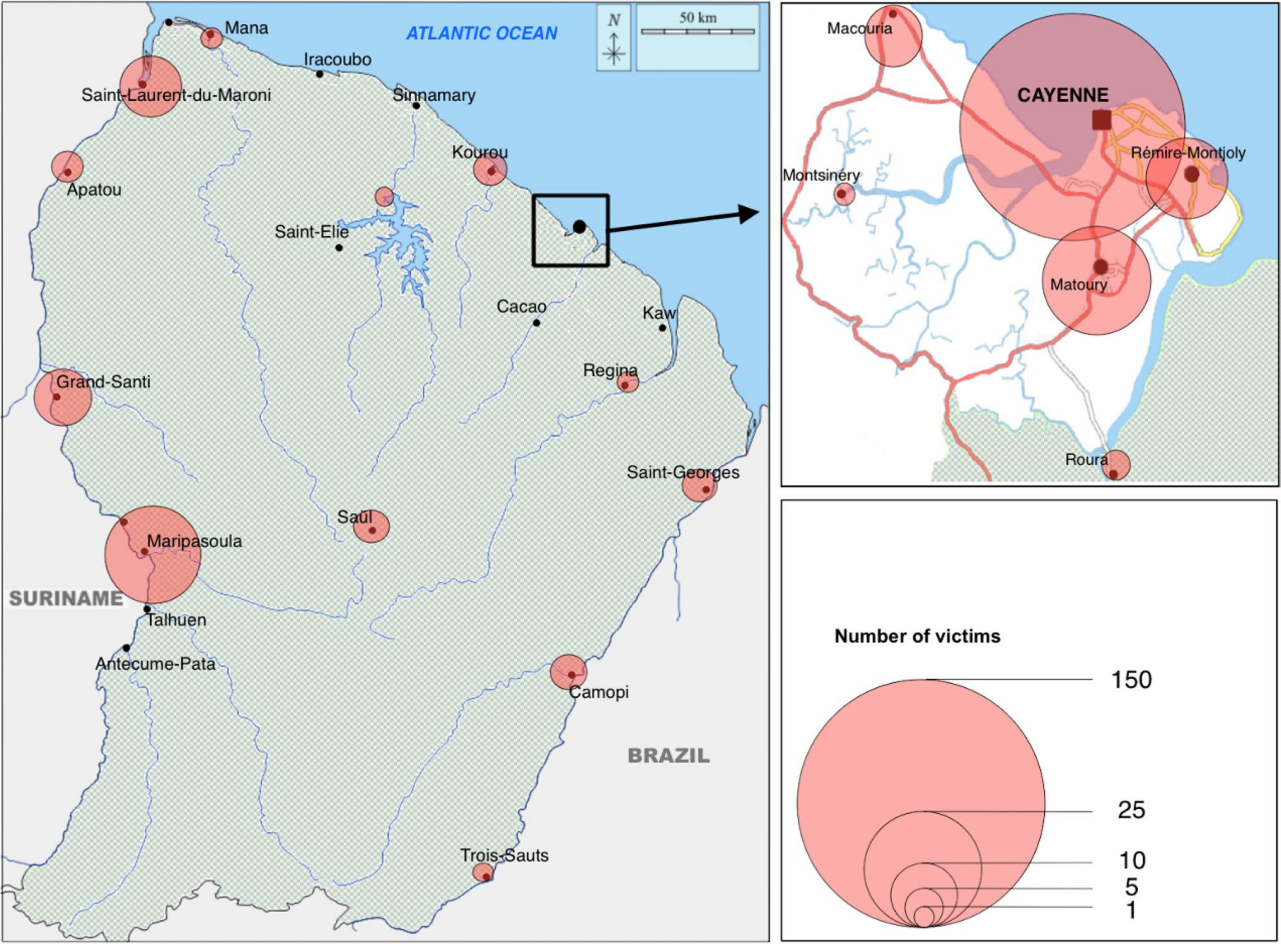
Geographic distribution of victims

We counted 101 victims including 22 (22%) deaths in 2016, 53 victims in 2017 including 14 (26%) deaths, 83 in 2018 including 17 (20%) deaths, and 102 in 2019 including 18 (18%) deaths. The geographic distribution of victims is presented in Fig. 2. The Cayenne agglomeration accounted for 233 (72%) of the trauma victims, including 170 (50%) cases in Cayenne.

### Management of victims

Over the 340 studied patients, 46 died without medical intervention, 4 died in other hospitals, and 290 victims were managed at the CGH. The overall mean length of hospital stay was 10 ± 18 days. A description of the management modalities is presented in Table 2. Over the 253 patients who received imaging, 15 (6%) had FAST ultrasounds and 146 (58%) had CT scans, and in 155 (61%) X-rays were performed. Regarding resuscitation maneuvers, 40 (14%) patients required abundant fluid infusion (≥ 1000 mL), 28 (10%) required blood transfusion, 26 (9%) required catecholamines, and 26 (9%) required mechanical ventilation. Finally, 117 (41%) required emergent surgery and 36 (12%) an admission in the intensive care unit. The average time from hospital admission to surgery was 9h16 ± 11h57. Return to home after emergency care was recorded in 117 patients (40%). The in-hospital mean length of stay was 17 ± 21 days, and the intensive care unit mean length of stay was 20 ± 32 days. There were 14 (5%) in-hospital deaths and 7 (2%) pre-hospital deaths.

**Table 2:** Description of patient’s management and post ED orientation

Variables	Victims managed in the CGH (*n* = 290) (mean or percentage)	CI 95% or SD
*Pre-hospital data*		
Call to EMS call center (*n* = 287)	175 (61%)	55–66%
EMS management (*n* = 286)	134 (47%)	40–52%
Incident—hospital admission delay (*n* = 195)	2h39	± 4h14
*Complementary exams*		
Blood test (*n* = 288)	199 (69%)	63–74%
Imaging	253 (87%)	83–91%
*Initial management*		
Resuscitation maneuvers (*n* = 287)	50 (17%)	13–22%
Antibiotic therapy (*n* = 286)	223 (78%)	73–83%
Blood transfusion (*n* = 285)	28 (10%)	7–14%
Emergent surgery (*n* = 288)	117 (41%)	35–46%
*Post ED orientation*		
Hospitalization	173 (60%)	54–65%
Intensive care unit admission	36 (12%)	9–17%
Death	21 (7%)	5–11%

*CGH* Cayenne General Hospital, *CI* confidence interval, *SD* standard deviation, *EMS* emergency medical service, *ED* emergency department

**Fig. 3:**
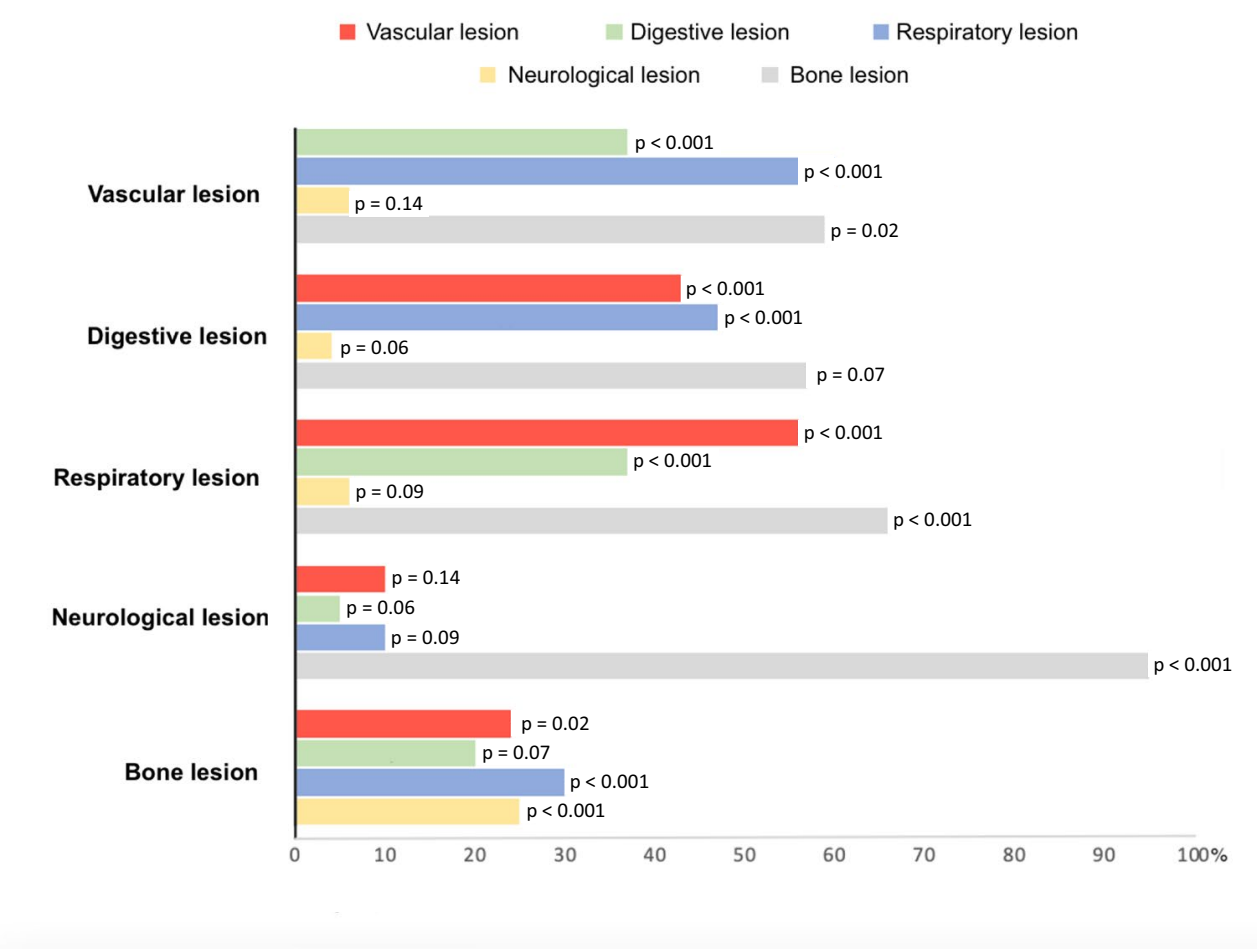
Associations between the injured anatomical sites

An analysis to identify the associations between the injured anatomical sites is detailed in Fig. 3. Bone injuries (*n* = 152) were statistically associated with central neurological (*n* = 40), vascular (*n* = 63), and respiratory (*n* = 68) injuries. There was also a strong association between vascular injuries and respiratory and digestive injuries (*n* = 53).

### Mortality and outcome

A comparison of survived versus died victims is presented in Table 3. Overall, mortality was estimated at 6.3/100,000 population per year. We counted 21 (7%) deaths among patients managed at the CGH. We did not find significant differences according to the type of weapon, the projectile used, or the presumed shooting distance. Additionally, the location of the event did not have an impact on mortality (coastal municipalities versus remote municipalities, *p* = 0.30). On the other hand, male gender (*p* = 0.026), daytime occurrence (*p* = 0.008), and suicides circumstances (*p* = 0.002) were significantly associated with death.

**Table 3:** Comparison of patient’s characteristics according to outcome

Variables	Surviving patients (*n* = 269)	Deceased patients (*n* = 71)	Odds ratio [IC 95%]	*p* value
*Demographic characteristics*				
Sex *(male)*	236 (88%)	70 (99%)	9.8 [2.05–175.6]	**0.026**
Age *(years)* (*n* = 334)	29 ± 11	33 ± 14	1.02 [1.00–1.05]	0.027
< 18 years old	26 (10%)	6 (9%)	0.93 [0.33–2.23]	0.88
*Incident circumstances*				
Day time (*n* = 221)				
Night (8:00 PM–8:00 AM)	118 (63%)	14 (40%)	0.38 [0.19–0.77]	**0.008**
Context (*n* = 318)				
Assault Accident Suicide Police arrest	209 (84%) 29 (12%) 5 (2%) 5 (2%)	56 (80%) 5 (7%) 8 (11%) 1 (1%)	1.5 [0.62–4.73] 0.64 [0.21–1.61] 9.3 [2.25–44.1] 1.16 [0.05–9.54]	0.38 0.38 **0.002** 0.79
Arm				
Long-barreled gun	56 (78%)	33 (92%)	3.14 [0.96–14.2]	0.074
Bullet (vs. pellets)	123 (50%)	31 (45%)	0.81 [0.47–1.38]	0.439
* Management*				
Incident—hospital admission delay	2h41 ± 4h19 (*n* = 184)	2h05 ± 2h42 (*n* = 12)	0 .99 [0.99–1.00]	0.635

**Fig. 4:**
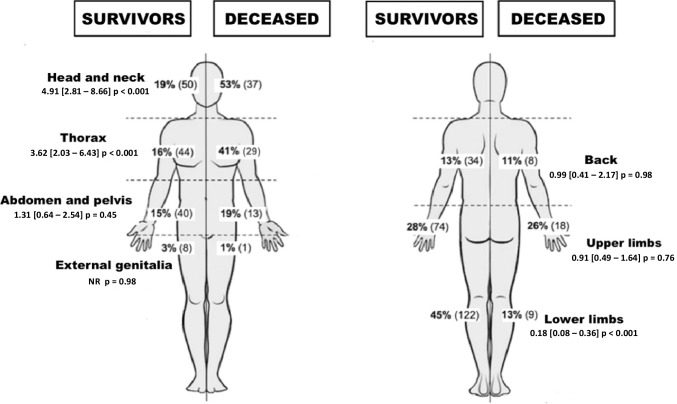
Sites of injuries compared to the survival status of patients (*n* = 340)

We also assessed the association between penetrating wound sites and mortality (Fig. 4): head and neck (*p* < 0.001) and thoracic involvement (*p* < 0.001) were associated with mortality. The presence of retained bullet fragments was also associated with death (84% *vs.* 65%, *p* = 0.003).

**Fig. 5:**
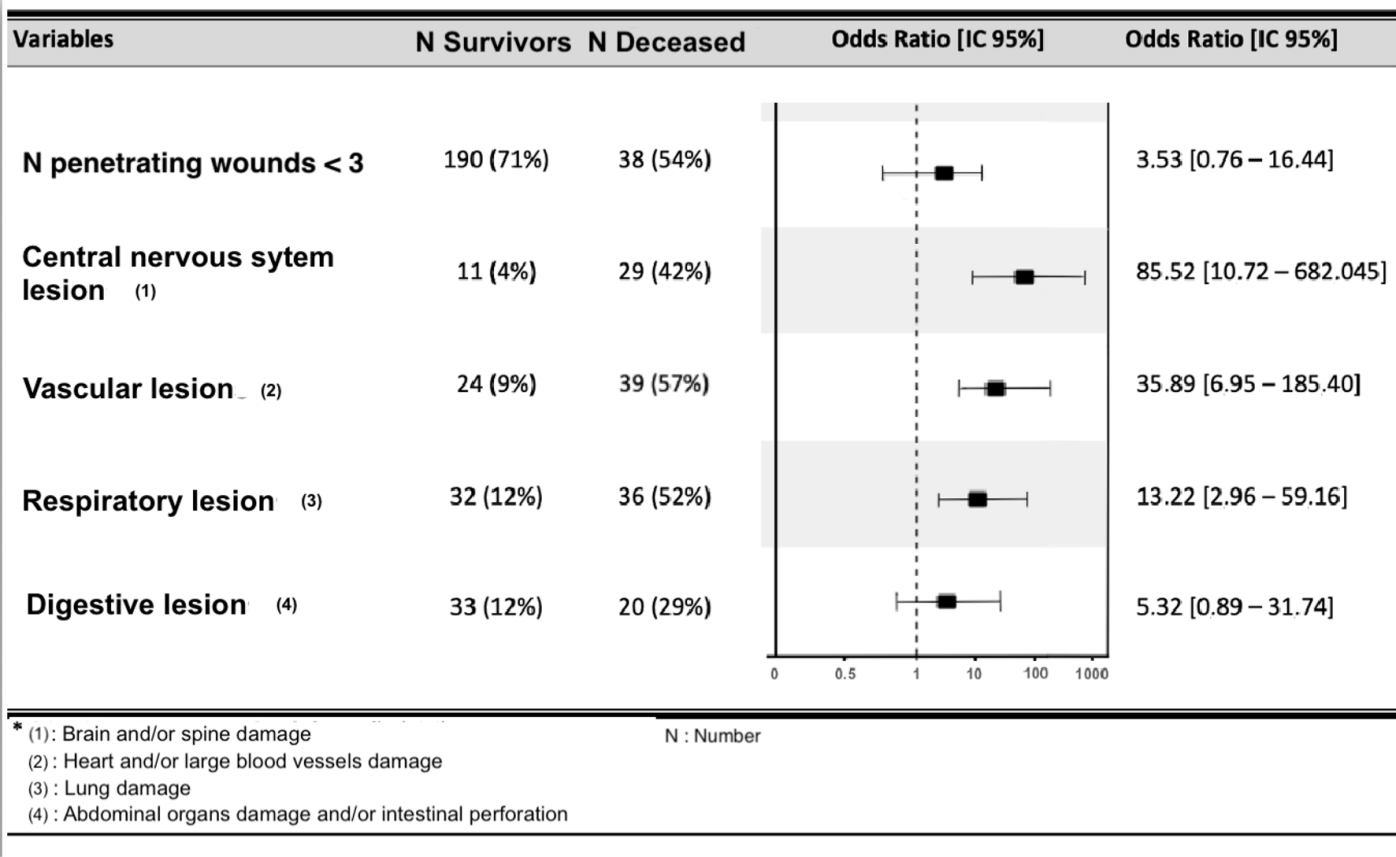
Multivariate analysis assessing the independent factors associated to mortality

Multivariate analyses assessing associated factors to death are shown in Fig. 5. Five variables selected by bivariate analysis and considered clinically relevant were used into the logistic regression model (number of wounds injury < 3, central nervous system injury, vascular injury, digestive injury, respiratory injury). Of these, three were significantly associated with an increased risk of death. Victims had an estimated relative risk of death 86 times and 36 times higher respectively when they had a central neurological injury (*p* < 0.001) or a vascular injury (*p* < 0.001). Respiratory injury showed a 13-fold higher odds ratio of death (*p* = 0.003).

## Discussion

This study describes the epidemiology of firearm injuries managed at the Cayenne General Hospital. This is the first study on the topic in French Guiana and one of the largest cohorts of civilian ballistic trauma victims in France. The population was predominantly young, male, and victim of assaults with a long-barreled gun. The population at risk found in our work but also circumstances are close to those found in the worldwide epidemiology of ballistic trauma [1]. This is all the more interesting given that 51% of the world’s firearm civilian deaths occur in six American countries, including some of French Guiana neighbors.

One of the points of this study was to describe the management of these patients. First of all, pre-hospital care by emergency medical service appears to be lower than in the USA (47% *vs.* 68%) [19]. Contrary to the Anglo-Saxon paramedics organization, in France, out-of-hospital emergency teams are organized on duty 24/7 and include doctors. For less severe injuries, teams of first rescuers not considered emergency medical service but with basic life support knowledge are mobilized. Regarding surgery, the proportion of surgical managements in our series is twice than in a US cohort study in 2021 [20]. This is probably due to our method including all patients needing emergency surgery compared to other studies excluding patients directly transferred to the emergency surgery room. We also note that the American studies mention a more frequent hospitalization rate (65% *vs.* 60%) as well as hospitalization in the intensive care unit (31% vs. 12%) [20]. In view of these results, it might seem that our population suffered less severe ballistic trauma than those reported in the literature. However, the mean length of stay in the intensive care unit is four times shorter in the USA (5 days *vs.* 20 days) than in our study.

Regarding mortality, the intra-hospital mortality rate was 4.8%, lower than in several American countries [1, 20]. The overall mortality (6.3/100,000 inhabitants/year) is significantly lower than results reported in the neighboring countries: 19.4/100,000 in Brazil and 10.6/100,000 in the USA but two to three times higher than the French national rate (2.7/100,000 inhabitants/year) [1]. These rates follow the overall crime rates and gun laws in the different countries. As found in previous works, our study identifies factors associated with an increased risk of death such as male gender or suicide context [1, 21]. In our results, deaths are associated with assaults in 80% and suicides in 11% of cases. Comparatively, in mainland France, suicide is the main cause of firearm-related deaths (79%) and concerns an older population [5]. In the USA, suicide accounts for 60% of firearm deaths [22]. The low suicide rate in our population could explain part of the difference about global mortality.

As described in the work of Maiden in 2009, who studied ballistics in firearm injuries, the study of wound sites and induced injuries finds a major impact on lethality [22]. In our series, head and/or neck and thorax injuries are associated with increased mortality. As found in a previous study, central neurological, respiratory, and vascular injuries are also associated with an increased risk of death [22, 23]. This finding is particularly interesting in terms of assessing the Cayenne Hospital’s technical facilities. Although these lesions can be very damaging and even life-threatening, requiring emergency surgery, stomatology, neurosurgery, and cardiothoracic surgery is under-represented or even absent in French Guiana [24, 25]. Induced mortality could be the direct cause of the absence of these specialties in our territory. In support of this hypothesis, abdominal-pelvic lesions, although frequently reported as fatal in the literature, are not so in our work, our hospital being adequately staffed with digestive surgeons [26].

Several areas for improvement emerge from this work in line to implement research on this topic insufficiently studied [27]. The most important issues accessible to primary prevention are violence and crime control, and gun laws: the population at risk being mainly young male victims of assault. According to Santaella-Tenorio’s study in 2016, gun ownership legislation as well as the number of weapons circulating is correlated with the number of related deaths [28]. Since 2017, the French Guiana Police Commissioner started to regulate the use and possession of firearms [16]. Despite its implementation in 2017, our work did not show a decrease in the number of firearm trauma victims during the study period. The high incidence of deaths from 2017 to 2019 follows the trend of violence and delinquency markers over the same period [12]. However, the continuous monitoring is necessary to evaluate the results of this new regulation in the medium and long term. This observation, as well as multiple proposals to fight violence, gold panning, and drug trafficking, is reported in the French Senate report in February 2020 [12].

CGH does not fill the characteristics of a “trauma center” due to a lack of some technical skills and facilities. There is also a large deficit in terms of intensive care capacity [29]. This aspect is responsible for long, costly, and often risky medical evacuations to specialized hospital centers in the French Caribbean islands or in mainland France [29]. The availability of interventional radiology techniques since 2020, the implementation of an observational database (TraumaBase®) in 2021, and the training of remote health center teams (university degrees: “severe trauma and life-threatening emergencies” and “initiation to emergency medicine”) since 2022 are the first responses for improving management of these patients [30–33].

Our study presents several limitations. First, this is a monocentric study. However, CGH is the referral and the main hospital in French Guiana with the majority of specialties, intensive care unit, and mobile emergency medical service. Second, this is a retrospective study with numerous missing data which leads to the classic biases of this methodology. The implementation of the TraumaBase® registry will allow a complete prospective data collection and a correct outcome evaluation of these traumas [31, 34]. Nevertheless, this study reports interesting results to understand the burden of firearm injuries in French Guiana. This work is a first step to adapt legislative texts according to the department context as it is discussed in the USA but also to improve medical care according to the national and international guidelines [35, 36].

## Conclusion

As reported in several American countries, this study highlights the significant incidence of ballistic trauma in French Guiana and the overexposure of young patients, victims of assaults with long-barreled guns. In spite of the geographical territory difficulties and the deficits in some technical procedures, the mortality remains low.

## Data Availability

The authors confirm that the data supporting the findings of this study are available on demand.

## References

[1] The Global Burden of Disease 2016 Injury Collaborators, Naghavi M, et al. Global mortality from firearms, 1990-2016. JAMA. 2018;320(8):792–814. 30167700 10.1001/jama.2018.10060 PMC6143020

[2] Alpers P, Wilson M. Impact global de la violence armée : Armes à feu et santé publique. Sydney School of Public Health, The University of Sydney ; 2014. Available on : https://www.gunpolicy.org/fr/firearms/region (Last access on Feb, 13^th^ 2023).

[3] Cerdà M. Editorial: Gun violence - risk, consequences, and prevention. Am J Epidemiol. 2016;183(6):516–7. 26865264 10.1093/aje/kwv327 PMC4782766

[4] Kravitz-Wirtz N, Bruns A, Aubel AJ, Zhang X, Buggs SA. Inequities in community exposure to deadly gun violence by race/ethnicity, poverty, and neighborhood disadvantage among youth in large US cities. J Urban Health. 2022;99(4):610–25. 35672546 10.1007/s11524-022-00656-0 PMC9172977

[5] DMCT, Unité traumatismes. Traumatismes par arme à feu en France métropolitaine : Données de mortalité (CépiDc 2000–2010), Enquête permanente sur les accidents de la vie courante (EPAC 2004–2011). Institut de veille sanitaire. 2013;Rapport TR13L029. Avaible on : https://armes-ufa.com/IMG/pdf/statistiques-2.pdf. Accessed 15 Feb 2024.

[6] Chanteur B, Reif X. Recensement de la population en Guyane : 276 128 habitants au 1er janvier 2018. Insee Flash Guyane, N°131; 2020 Dec. Available on : https://www.insee.fr/fr/statistiques/5005684 (Last access on Feb, 13th 2023).

[7] Duplan H, Sanna A, Rousseau C, De Bort C. Focus. Géographie, démographie et offre de soins en Guyane. Bull Epidemiol Hebd. 2020 Dec; (36–37):698–702.

[8] Pujo JM, Mutricy R, Kraiem H, et al. Telemedicine in French Guiana: implementation et emergency care perspective. J Int Soc Telemed eHealth. 2021;9:e6.

[9] Agouram S. Le CHAR : trauma center malgré lui ? [Medicine Thesis] University of the French Antilles, France; 2018. Available on : https://dumas.ccsd.cnrs.fr/dumas-02265870/document. Accessed 13 Feb 2023.

[10] Naulin A. De nombreuses victimes de délinquance d’appropriation et de violences en Guyane. Insee Analyses Guyane, N°20; 2017 Jan. Available on : https://www.insee.fr/fr/statistiques/2565363 (Last access on Feb, 13^th^ 2023).

[11] Fremery A, Piriou V, Bonnefoy C, et al. Description and evaluation of cocaine body-packers management in French Guiana. J Forensic Leg Med. 2023;95:102500. 36827732 10.1016/j.jflm.2023.102500

[12] Bas P, Darnaud M, Fichet JL, Joissains S, Mohamed Soilihi T. Pour une grande loi Guyane : 52 propositions. SENAT. 2020 Feb, 337;127p. Available on : https://www.senat.fr/rap/r19-337/r19-337.html (Last access on Feb, 13^th^ 2023).

[13] Ministère de l’Intérieur. La délinquance enregistrée outre-mer : des situations très variées selon les territoires. Interstats 2016 May. Available on : https://www.interieur.gouv.fr/Interstats/Actualites/Info-rapide-n-5-La-delinquance-enregistree-outre-mer-des-situations-tres-variees-selon-les-territoires (Last access on Feb, 13th 2023).

[14] SSMSI. Insécurité et délinquance en 2018 : premier bilan statistique. Interstats. 2019; 3:82–191. Available on : https://www.interieur.gouv.fr/Interstats/Actualites/Insecurite-et-delinquance-en-2018-premier-bilan-statistique (Last access on Feb, 13^th^ 2023).

[15] Nacher M, Deungoue S, Brousse P, Adenis A, Couppié P, Sobesky M. Calcul de l’IP-DMS en Guyane : prendre en compte le poids réel de la précarité et de l’isolement. Rev Epidemiol Sante publique. 2020;68(2):125–32. 32035728 10.1016/j.respe.2019.09.012

[16] Préfet de la région Guyane. Arrêté du 23 mai 2016 réglementant l’usage des armes à feu dans le département de la Guyane. DRCI. 2016 May 23; 48–50. Available on : https://guyane.ofb.fr/wp-content/uploads/2018/04/AP-du-23-mai-2016-r%C3%A9glementant-lusage-des-armes-%C3%A0-feu-dans-le-d%C3%A9partement-de-Guyane.pdf (Last access on Feb, 13^th^ 2023).

[17] Gastineau KAB, McKay S. Firearm injury prevention. Pediatr Clin North Am. 2023;70(6):1125–42. 37865435 10.1016/j.pcl.2023.07.003

[18] Richmond TS, Foman M. Firearm violence: a global priority for nursing science. J Nurs Scholarsh. 2019;51(3):229–40. 30215887 10.1111/jnu.12421 PMC6417970

[19] Masmejean EH, Faye A, Alnot JY, Mignon AF. Trauma care systems in France. Injury. 2003;34(9):669–73. 12951291 10.1016/s0020-1383(03)00146-3

[20] Asmar S, Bible L, Vartanyan P. Firearm-related injuries: a single center experience. J Surg Res. 2021;265:289–96. 33964639 10.1016/j.jss.2021.03.058

[21] Lichte P, Oberbeck R, Binnebösel M, Wildenauer R, Pape HC, Kobbe P. A civilian perspective on ballistic trauma and gunshot injuries. Scand J Trauma Resusc Emerg Med. 2010;18:35. 20565804 10.1186/1757-7241-18-35 PMC2898680

[22] Centers for Disease Control and Prevention. Leading causes of death reports, national and regional, 2010–2020. Available on : https://wisars.cdc.gov/data/explore-data/ (Last access on April, 10th 2023).

[23] Maiden N. Ballistics reviews: mechanisms of bullet wound trauma. Forensic Sci Med Pathol. 2009;5(3):204–9. 19644779 10.1007/s12024-009-9096-6

[24] Breeze J, Powers DB. Current opinion in the assessment and management of ballistic trauma to the craniomaxillofacial region. Curr Opin Otolaryngol Head Neck Surg. 2020;28(4):251–7. 32520756 10.1097/MOO.0000000000000634

[25] Galbraith CM, Wagener BM, Chalkias A, Siddiqui S, Douin DJ. Massive trauma and resuscitation strategies. Anesthesiol Clin. 2023;41(1):283–301. 36872005 10.1016/j.anclin.2022.10.008 PMC10688568

[26] Durso AM, Paes FM, Caban K, Danton G, Braga TA, Sanchez A, Munera F. Evaluation of penetrating abdominal and pelvic trauma. Eur J Radiol. 2020;130:109187. 32745896 10.1016/j.ejrad.2020.109187

[27] Donnelly KA, Kafashzadeh D, Goyal MK, Badolato GM, Patel SJ, Bhansali P, Roche KM, Cohen JS. Barriers to firearm injury research. Am J Prev Med. 2020;58(6):825–31. 32147369 10.1016/j.amepre.2020.01.005

[28] Santaella-Tenorio J, Cerdá M, Villaveces A, Galea S. What do we know about the association between firearm legislation and firearm-related injuries? Epidemiol Rev. 2016;38:140–57. 26905895 10.1093/epirev/mxv012 PMC6283012

[29] Kallel H, Resiere D, Houcke S, et al. Critical care medicine in the French Territories in the Americas: current situation and prospects. Rev Pana Salud Publica, 2021 10.26633/RPSP.2021.46 PMC8080944 33936184

[30] Nyberger K, Caragounis EC, Djerf P, Wahlgren CM. Management and outcomes of firearm-related vascular injuries. Scand J Trauma Resusc Emerg Med. 2023;31(1):35. 37420263 10.1186/s13049-023-01098-6 PMC10327304

[31] Moyer JD, Hamada SR, Josse J, Auliard O, Gauss T, Traumabase Group. Trauma reloaded: trauma registry in the era of data science. Anaesth Crit Care Pain Med. 2021;40(2):100827. 33741513 10.1016/j.accpm.2021.100827

[32] Descamps C, Hamada S, Hanouz JL, et al, Traumabase Group. Gunshot and stab wounds in France: descriptive study from a national trauma registry. Eur J Trauma Emerg Surg. 2022;48(5):3821–9. 34232339 10.1007/s00068-021-01742-9

[33] Fremery A, Blanc R, Mutricy R, Kallel H, Pujo JM. Physicians’ perceptions of life-threatening emergency management in Guyanese health centers. Ann Fr Med Urgence. 2022;12:219–23.

[34] Houwert RM, Balogh ZJ, Lefering R. Trauma registries: towards global standardisation and outcome evaluation. Eur J Trauma Emerg Surg. 2023;49(4):1611–2. 37555992 10.1007/s00068-023-02332-7

[35] Gonzalez-Guarda RM. Applying lessons from major public health accomplishments to firearm injuries in the US. JAMA Health Forum. 2023;4(6):e232201. 37261834 10.1001/jamahealthforum.2023.2201

[36] Mueller KL, Lovelady NN, Ranney ML. Firearm injuries and death: a United States epidemic with public health solutions. PLOS Glob Public Health. 2023;3(5):e0001913. 37224135 10.1371/journal.pgph.0001913 PMC10208504

